# Impact of Reducing the Procedure Time on Thromboembolism After Coil Embolization of Cerebral Aneurysms

**DOI:** 10.3389/fneur.2018.01125

**Published:** 2018-12-18

**Authors:** Sang-Hwa Lee, Min Uk Jang, Jihoon Kang, Yeo Jin Kim, Chulho Kim, Jong-Hee Sohn, Jinseo Yang, Jin Pyeong Jeon, Yongjun Cho, Hyuk Jai Choi

**Affiliations:** ^1^Department of Neurology, Hallym University College of Medicine, Chuncheon Sacred Heart Hospital, Chuncheon, South Korea; ^2^Department of Neurology, Hallym University College of Medicine, Dongtan Sacred Heart Hospital, Hwaseong, South Korea; ^3^Department of Neurology, Seoul National University Bundang Hospital, Seoul National University, Seongnam, South Korea; ^4^Department of Neurosurgery, Hallym University College of Medicine, Chuncheon Sacred Heart Hospital, Chuncheon, South Korea

**Keywords:** thromboembolism, coil embolization, cerebral aneurysm, procedure-related factors, procedure time

## Abstract

**Background:** There is still controversy regarding which procedure-related factors affect the occurrence of periprocedural thromboembolism. This study aimed to investigate which procedure-related risk factors can be modified to prevent adverse thromboembolic events after coil embolization of intracranial aneurysm.

**Methods:** Using a single-center database, we retrospectively identified a consecutive series of patients with symptomatic and asymptomatic cerebral aneurysms treated with coil embolization. We evaluated the following procedure-related factors: procedure time, procedure methods (simple coiling, stent-assisted coiling, and use of multiple microcatheters), and number of coils inserted. The primary outcome was the development of thromboembolism before and after coil embolization confirmed by diffusion-weighted imaging (DWI) irrespective of the location of the procedure. Pearson's chi-square, Student's *t*-test, multivariable logistic regression analysis, and sensitivity analysis with multinomial logistic regression analysis were used in the statistical analyses.

**Results:** Of 180 cases enrolled, 146 (81.1%) had evidences of thromboembolism confirmed by DWI, and 13 (7.2%) had neurologic symptoms. Among the documented modifiable procedure-related factors, every 10 min increase in the procedure time was independently associated with the risk of thromboembolism, after adjusting the analysis (adjusted odds ratio 1.11; 95% confidence interval 1.01–1.21). The coiling methods, use of multiple catheters, and number of coils inserted did not change the effect of the procedure time on thromboembolic events (*p* for interactions > 0.05).

**Conclusion:** This study showed that the procedure time might be the most effective modifiable factor for reducing thromboembolic events irrespective of the procedure methods used during coil embolization of cerebral aneurysms.

## Introduction

The incidence of periprocedural thromboembolism is reported as 10–69%, and it is the most frequent complication of coil embolization for cerebral aneurysm (1–11). Since periprocedural thromboembolism immediately causes significant neurological deficit and increases the risk of delayed cognitive decline even in immediate asymptomatic lesions, it would be of interest to determine its risk factors and to develop preventative strategies (12, 13).

Previous evidences have shown that some factors, such as age and aneurysmal size, are important risk factors for periprocedural thromboembolism (2, 4, 9, 11). Age and aneurysmal size are non-modifiable factors so they would be helpful only to distinguish the patients at high risk of periprocedural thromboembolism.

There is still controversy regarding the procedure-related factors, although the procedure technique itself is considered to have a high possibility of affecting the occurrence of periprocedural thromboembolism. One explanation for this is that prior studies assessed the procedure technique, which was mostly a newly introduced coil device or technique, so it was difficult to evaluate the effects of various risk factors. Second, some of the previous studies considered the effect of the treating physician a risk (1, 3, 5, 14).

This study aimed to investigate which risk factors can be modified to prevent periprocedural thromboembolism in coil embolization for cerebral aneurysm by using a single interventionist's consecutive series of patients.

## Methods

### Study Population

We prospectively registered all cases with intracranial aneurysms hospitalized between January 2013 and March 2017 in our institution. In this study, we retrospectively collected only cases with intracranial aneurysm who underwent magnetic resonance imaging (MRI) before and within 48 h after coil embolization was performed by an expert interventionist (HC). The following cases were excluded from this study: (1) patients who were uncooperative or unavailable for MRI because of medical conditions or neurological disability; (2) patients who were documented as having an atherosclerotic lesion located proximal to the aneurysm site on the pre-procedure or post-procedure MRI study. This was meant to exclude biologic factors that have a possibility of affecting ischemic lesions during the procedure.

### Coil Embolization Procedure

All procedures were performed under general anesthesia using a biplane angiography system (Siemens Medical System, Erlangen, Germany). After a 7-French guiding catheter was placed into the internal carotid or vertebral artery, a microcatheter was introduced into the aneurysmal sac with a microwire (Synchro-14, Boston Scientific Corporation, Natick, MA). The types of endovascular procedures were divided into two groups: simple coiling and stent-assisted coil embolization. Stent-assisted coil embolization was performed by an interventionist as an adjunctive technique in consideration of various patient and aneurysmal factors. For simple coiling, multiple microcatheters were only used for aneurysms with broad necks or incorporated arterial branches. Anticoagulation therapy with intravenous heparin (3,000 units) was administered after guided catheter placement, followed by intermittent hourly heparin injections (1,000 units).

### Definition of Parameters

Acute thromboembolisms after coil embolization were defined by the lesions with high signals on diffusion-weighted imaging (DWI) that correlated with low signals on an ADC map. Two experienced vascular neurologists (S-HL and MJ) reviewed all DWI studies and collected formal MRI readings to detect acute thromboembolism. The volume of the thromboembolic lesion confirmed by DWI within 48 h after coil embolization was calculated using Medical Image Processing and Visualization software (version 7.3.0, National Institutes of Health, Bethesda, MD).

From the registry database, we directly obtained patients' demographics (age and sex), vascular risk factors, and medical history (hypertension, diabetes mellitus, hyperlipidemia, and coronary artery disease; current smoking status; history of stroke; and prior use of antithrombotic agents). The aneurysm size, location, and morphology (fusiform and saccular) were noted. Premedication with antiplatelet agents before coil embolization was also noted.

The modifiable procedure-related factors were defined as the procedure time, simple coiling, stent-assisted coiling, use of multiple microcatheters, and number of coils inserted, which were documented in previous studies (10, 11, 14–16). The procedure time was defined as the time from groin puncture to guiding catheter removal. Use of multiple microcatheters was defined as using more than a single microcatheter which indicates multiple manipulations of the aneurysm and delayed occlusion time during coil embolization regardless of the coiling method used (10, 17).

The primary outcome measure was the development of thromboembolisms confirmed by DWI before and after coil embolization. The secondary outcome measure was defined as three DWI groups: the DWI-negative lesion group, coiling-related DWI-positive lesion group, and coiling-unrelated DWI-positive lesion group. The coiling-related DWI-positive lesions were ipsilateral lesions located in the relevant territory of the coiling site. The coiling-unrelated DWI-positive lesions were contralateral or remote lesions located in an irrelevant territory of the coiling site.

### Statistical Analysis

Summary statistics are presented as mean ± standard deviation or frequency (percentage) as appropriate. Baseline characteristics were compared according to the occurrence of thromboembolism using Pearson's chi-square test for categorical variables and Student's *t* test for continuous variables as appropriate. To evaluate the clinical environment of procedure-related factors, we described the frequency of each procedure-related factor for thromboembolisms by bivariate analysis. Additionally, mediator effect analysis was performed to determine the association between each procedure-related factor and the occurrence of thromboembolism.

Multivariable logistic regression analysis was used to determine the individual risk of each procedure-related factor for thromboembolism. Variables for adjustment in multivariable models were selected if their *p*-values were <0.1 in crude comparisons or clinically plausible. Moreover, we evaluated the association between procedure-related factors and the DWI-negative lesion group, coiling-related DWI-positive lesion group, and coiling-unrelated DWI-positive lesion group using sensitivity analysis with multinomial logistic regression analysis.

## Results

Among 159 subjects (62 men, 97 women; mean age 57.1 ± 13.4 years), 180 cases were enrolled. Sixty-six patients (42%) had hypertension, 17 (11%) had diabetes mellitus, 6 (4%) had coronary artery disease, 10 (6%) had hyperlipidemia, 48 (30%) were current smokers, and 32 (20%) had a history of stroke. Among 180 aneurysms, 109 (61%) were ruptured. Sizes of the aneurysms were as follows: ≤ 7 mm, 140 (77.8%); and >7 mm, 40 (22.2%). The distributions of aneurysms according to their location were as follows: middle cerebral artery, 20 (11.1%); anterior cerebral artery, 72 (40.0%); internal carotid artery, 69 (38.3%); and posterior circulation artery (basilar artery and vertebral artery), 19 (10.6%).

The overall rate of thromboembolism confirmed by DWI after coil embolization was 81.1% (*n* = 146). Among the 146 DWI-positive lesions, 13 (8.9%) were symptomatic, and 133 (91.1%) were asymptomatic. Among the 13 patients with symptomatic DWI-positive lesions, 7 patients had dependent ambulation (modified Rankin Scale [mRS] score of 0 to 2), but 6 patients experienced poor functional status at hospitalization (3 had an mRS score of 4, and 3 had an mRS score of 5). All 6 patients with poor functional status had a ruptured aneurysm, and among 7 patients with an mRS score of 0 to 2, 6 patients had an unruptured aneurysm (Supplemental Figure 1). The patients with thromboembolism after coil embolization were more likely to be hypertensive (*p* < 0.05), have a longer procedure time (*p* < 0.05), and be older (*p* = 0.06). The frequencies of patient and aneurysmal factors were not different between patients with and without thromboembolism (Table 1). Among 146 cases of DWI-positive lesions, 4 cases of vasospasms occurred during coil embolization. An unfavorable in-hospital functional outcome, defined by a modified Rankin Scale score >1, was significantly higher in patients with thromboembolism than in those without thromboembolism (32.2% vs. 11.8%, *p* = 0.01).

**Table 1:** Comparison of baseline characteristics according to the presence of thromboembolism after coil embolization.

	**Thromboembolism (–) *n* = 34**	**Thromboembolism (+) *n* = 146**	***p*-value**
**PATIENT FACTORS**			
Age (year), SD	50.8 ± 11.2	58.6 ± 13.4	0.06^‡^
Male sex, %	10 (29.4)	57 (39.0)	0.33^*^
Hypertension, %	7 (20.6)	69 (47.3)	0.003^*^
Diabetes mellitus, %	2 (5.9)	17 (11.6)	0.54^†^
Coronary artery disease, %	0 (0.0)	7 (4.8)	0.35^†^
Hyperlipidemia, %	2 (5.9)	10 (6.8)	0.60^†^
Current smoker, %	10 (29.4)	41 (28.1)	0.88^*^
History of stroke, %	12 (35.3)	39 (26.7)	0.40^*^
History of using antiplatelet agents, %	5 (14.7)	18 (12.3)	0.78^†^
**ANEURYSMAL FACTORS**			
Size of the aneurysm			0.10^*^
≤7 mm	26 (76.5)	114 (78.1)	
>7 mm	8 (23.5)	32 (21.9)	
Ruptured aneurysm, %	22 (64.7)	87 (59.6)	0.70^*^
Aneurysm location, %			0.95^*^
MCA	3 (8.8)	17 (11.6)	
ACA	15 (44.1)	57 (39.0)	
ICA	13 (38.2)	56 (38.4)	
BA and VA	3 (8.8)	16 (11.0)	
Morphology of the aneurysm, %			0.81^*^
Fusiform	8 (23.5)	31 (21.2)	
Saccular	26 (76.5)	115 (78.8)	
**PROCEDURE-RELATED FACTORS**			
Simple coiling, %	17 (50.0)	73 (50.0)	1.00^*^
Stent-assisted coiling, %	13 (38.2)	46 (31.5)	0.54^*^
Use of multiple microcatheters, %	4 (11.8)	27 (18.5)	0.45^*^
Number of coils inserted (*n*), SD	4.9 ± 3.5	4.8 ± 4.6	0.70^‡^
Procedure time (min), SD	92.7 ± 37.2	114.7 ± 63.7	0.01^‡^
Premedication with antiplatelet agents, %	14 (41.2)	65 (44.5)	0.85^*^
**LABORATORY RESULTS**			
Hemoglobin level (mg/dl), SD	13.4 ± 1.6	15.4 ± 15.1	0.30^‡^
Platelet count (×1,000/u), SD	266.7 ± 86.9	261.2 ± 75.5	0.36^‡^
Total cholesterol level (mg/dl), SD	171.7 ± 30.5	180.2 ± 41.4	0.11
**CLINICAL OUTCOMES**			
Symptomatic events, %	0 (0.0)	13 (8.9)	0.13^†^
Discharge mRS score, %			0.01^*^
≤1	30 (88.2)	99 (67.8)	
>1	4 (11.8)	47 (32.2)	

*SD, standard deviation; MCA, middle cerebral artery; ACA, anterior cerebral artery; ICA, internal carotid artery; BA, basilar artery; VA, vertebral artery; mRS, modified Rankin Scale*.

* *Calculated by the chi-square test*.

† *Calculated by Fisher's exact test*.

‡ *Calculated by Student's t-test*.

Multivariable analysis showed that a longer procedure time increased the risk of thromboembolism (Table 2). The other procedure-related factors were not statistically associated with thromboembolism after coil embolization. The degree of multicollinearity using variance inflation (VIF) factor was not significant between the procedure time and other procedure-related factors (all, VIF < 1.5). The mediator effect showed that the association between the procedure time and occurrence of thromboembolism was not significantly modified by any of the other documented procedure-related factors (Table 3).

**Table 2:** Logistic regression analysis^*a*^ showing ORs of each procedure-related factor for the occurrence of thromboembolism.

	**Adjusted OR**	**95% CI**
Age	1.05	1.02–1.09
Hypertension	2.88	1.09–7.59
Aneurysmal size	1.48	0.53–4.12
Procedure time per 10 min increase	1.11	1.01–1.21
Simple coiling	0.79	0.21–2.96
Use of multiple microcatheters	1.27	0.34–4.80
Stent-assisted coiling	0.34	0.09–1.31
Number of inserted coils	0.95	0.84–1.06

a *The logistic regression analysis was adjusted for age, hypertension, aneurysmal size, and each procedure-related factor separately*.

*ORs, odds ratios; CI, confidence interval*.

**Table 3:** Interaction analysis showing which procedure-related factors mediated the effect of procedure time on the occurrence of thromboembolism.

	***p* for interaction**
Effect of simple coiling	0.23
Effect of use of multiple microcatheters	0.87
Effect of stent-assisted coiling	0.30
Effect of the number of coils inserted	0.88

*All models were adjusted for age, hypertension, aneurysmal size, and each procedure-related factor. None of the procedure-related factors' change of significance was associated between the procedure time and occurrence of thromboembolism*.

In the *post-hoc* analysis, we compared the remaining procedure-related factors according to the procedure time, which was the only significant variable for evaluating the periprocedural environment of coil embolization. With the respect to the dichotomized procedure time (≥120 min vs. <120 min), the group with the longer procedure time used more devices, the stent-assisted coiling method, and multiple microcatheters and coils (Table 4). Patient-related and aneurysm-related factors were not different whether or not the procedure time was delayed. The volume of the thromboembolism confirmed by DWI significantly increased with an increasing procedure time (*p* = 0.04).

**Table 4:** Comparison of baseline characteristics according to the procedure time in patients treated with coil embolization.

	**Procedure time ≤120 min *n* = 121**	**Procedure time >120 min *n* = 59**	***p*-value**
**PATIENT FACTORS**			
Age (year), SD	57.0 ± 12.7	57.5 ± 14.9	0.22^‡^
Male sex, %	47 (38.8)	20 (33.9)	0.62^*^
**VASCULAR RISK FACTORS**			
Hypertension, %	51 (42.1)	25 (42.4)	0.98^*^
Diabetes mellitus, %	12 (9.9)	7 (11.9)	0.80^*^
Coronary artery disease, %	5 (4.1)	2 (3.4)	0.58^†^
Hyperlipidemia, %	11 (9.1)	1 (1.7)	0.11^†^
Current smoker, %	38 (31.4)	13 (22.0)	0.22^*^
History of stroke, %	34 (28.1)	17 (28.8)	0.92^*^
History of using antiplatelet agents, %	14 (11.6)	9 (15.3)	0.64^*^
**ANEURYSMAL FACTORS**			
Size of the aneurysm, %			0.97^*^
≤7 mm	94 (77.7)	46 (78.0)	
>7 mm	27 (22.3)	13 (22.0)	
Ruptured aneurysm, %	74 (61.2)	35 (59.3)	0.87^*^
Aneurysm location, %			0.99^*^
MCA	14 (11.6)	6 (10.2)	
ACA	48 (39.7)	24 (40.7)	
ICA	46 (38.0)	23 (39.0)	
BA and VA	13 (10.7)	6 (10.2)	
Morphology of the aneurysm, %			0.85^*^
Fusiform	27 (22.3)	12 (20.3)	
Saccular	94 (77.7)	47 (79.7)	
**PROCEDURE-RELATED FACTORS**			
Simple coiling, %	74 (61.2)	16 (27.1)	<0.001^*^
Stent-assisted coiling. %	33 (27.3)	26 (44.1)	0.03^*^
Use of multiple microcatheters, %	14 (11.6)	17 (28.8)	0.01^*^
Number of coils inserted, n	4.7 ± 3.4	5.2 ± 6.0	0.03^‡^
Premedication with antiplatelet agents, %	47 (38.8)	32 (54.2)	0.06^*^
Lesion volume (mm^3^), IQR	110.2 (21.5–1273.7)	150.1 (50.3–7737.1)	0.04^§^

*SD, standard deviation; MCA, middle cerebral artery; ACA, anterior cerebral artery; ICA, internal carotid artery; BA, basilar artery; VA, vertebral artery; DWI, diffusion-weighted imaging; IQR, interquartile range*.

* *Calculated by the chi-square test*.

† *Calculated by Fisher's exact test*.

‡ *Calculated by Student's t-test*.

§ *Calculated by the Mann-Whitney test*.

Coiling-related thromboembolism seemed to have an increasing trend with an increasing procedure time (Supplemental Table 1). The various coiling methods and devise used (single or stent assisted coiling or use of multiple microcatheters and number of coils inserted) were not significantly different with coiling-related and coiling-unrelated thromboembolisms. The sensitivity analysis with multinomial logistic regression analysis showed that an increased procedure time also increased the occurrence of coiling-related thromboembolism, whereas it did not increase the occurrence of coiling-unrelated thromboembolism (Supplemental Table 2).

## Discussion

This study aimed to examine which modifiable procedure-related factors could be effective to reduce thromboembolism in coiling embolization of cerebral aneurysm. It has been reported that several procedure-related factors could affect thromboembolic events after coil embolization. However, these factors focused on novel devices and improving the individual technique and workmanship of the interventionist. From the perspective of a clinical neurologist who refers to an interventionist to treat an aneurysm, practicable efforts to determine effective factors that reduce thromboembolic events may be considered more important than improving the individual interventionist's workmanship and technique. Considering above mentioned, this study had main findings as follows; (1) we found that among the modifiable procedure-related factors, reducing the procedure time could be effective for reducing the risk of thromboembolism after coil embolization; (2) we also suggested that practical efforts, such as reducing procedure manipulations and establishing efficient procedure process, should be performed by the interventionist to reduce the procedure time in real-world practice.

The procedure time as a risk factor for thromboembolism has been rarely documented in previous studies (11). Herein, thromboembolism after coil embolization tended to increase with an increasing procedure time, but this was not found with the patient and aneurysmal factors. The volume of the thromboembolism confirmed by DWI also tended to increase with an increasing procedure time. In addition, we found that the interventionist had a longer procedure time with the use of multiple microcatheters and many devices, whereas this was not the case with the aneurysmal size, morphologies of the aneurysm, and patients' emergent status. These findings mean that many procedural manipulations during coil embolization (14) could lead to delayed intra-procedural time, thereby increasing thromboembolism. Hence, when considering various coiling techniques according to the patients' and aneurysmal status before a procedure plan is established, interventionists should keep in mind that the procedure time could be an effective factor for reducing thromboembolisms.

Multivariable analysis also showed that reducing the procedure time could be the most effective procedure-related factor for reducing thromboembolisms during coil embolization. The insignificance of ORs for the other procedure-related factors was consistent with the result of a recent previous study (16). Notably, mediator effect analysis showed that the other procedure-related factors could not be attributable to the procedure time and thromboembolisms after coil embolization. This finding could support our main practical finding that the procedure time could still be considered an effective factor for reducing thromboembolism compared to other technical and device-related factors. Additionally, the positive association between the procedure time and coiling-related thromboembolism in sensitivity analysis supported the robustness of our main findings.

Interestingly, the finding that the procedure time was independent from other procedure-related factors by the degree of multicollinearity could explain which factors mainly extend the procedure time. A previous study showed that a long procedure time could be associated with the use of a device, aneurysmal size, and procedural morbidity (14). This previous study did not evaluate independency between the procedure time and other factors. With the respect to the result of multicollinearity, we carefully speculated that inefficient periprocedural environment, besides documented procedure-related factors, could contribute to increasing the procedure time of coil embolization in real-world practice. However, since the inefficient periprocedural factors that increased the procedure time were unavailable in our database, we carefully suggested that those factors could be uncooperative and inexperienced coworkers, an inefficient procedure process (e.g., lack of proficiency of the nursing work force, expertise of paramedics and technicians and any of unsupportive external factors to hinder expedient and efficient coil embolization) and unexpected situations (e.g., unstable vital signs of the patients, the occurrence of procedural rupture and procedural morbidity, defined as complications leading to death, and permanent neurological disability) during coil embolization (14) These findings could help interventionists establish a more efficient procedural process of coil embolization to reduce the procedure time.

Our findings with overall rates of 81.1% for thromboembolism and 7.2% (based on 13/180) for symptomatic events showed that the occurrence of thromboembolism after coil embolization for cerebral aneurysm remained high, but the rate of symptomatic events after coil embolization was reasonable compared to that reported in previous studies (range from 5.4 to 40%) (4, 6, 8). Nonetheless, 32.2% of patients with thromboembolism confirmed by DWI had an unfavorable in-hospital functional outcome, mRS score >1. Among the patients with an unruptured aneurysm, the rates of symptomatic events and unfavorable in-hospital functional outcome were 10.2 and 18.6%, respectively (data not shown). We found no studies that described the aggravating functional status in coil-treated patients who had thromboembolism during hospitalization. The previous studies just emphasized the safety of the coiling procedure since most of the DWI-positive lesions were asymptomatic (1, 18). Although the preprocedural mRS scores were unavailable in our database, the rate of an unfavorable in-hospital functional status after coil embolization in patients with unruptured aneurysm leads to an awakened interest in efforts to reduce thromboembolism after the endovascular procedure.

Our study has several limitations. First, it was a retrospective, observational study with a single-center registry, although we used a prospective database. Second, since an experienced interventionist performed the endovascular procedures, selection bias could affect the results, despite use of a consistent procedural protocol. However, our intentions were to describe the clinical periprocedural situation in the real world and determine the most modifiable factor for reducing a complication of coil embolization, not to evaluate the safety and effectiveness of coil embolization. Hence, in determining the impact of procedure-related factors on outcome, it could be considered a strength of this study that only patients treated by one interventionist were enrolled with consistent coiling protocol. Third, procedure-related factors determined in our study cannot be representative of the general population, and unmeasured confounding factors were not controlled for, although we controlled for known measurable confounders in the multivariable model. Therefore, we should be cautious in generalizing the study results. Lastly, we could not demonstrate the inefficient periprocedural factors that delay the procedure time in detail. Additionally, we did not provide the standard procedure time in this study. The mean procedure time in this study was 110.6 min (± 60.2 min), which was longer than that in a previous study (14). However, our intention in this study was to determine the modifiable factor for reducing thromboembolism after coil embolization, not to define the standard procedure time. Although we did not determine the “gold standard procedure time” for reducing thromboembolisms, our study findings may increase interventionists' interest in improving the procedural process.

## Conclusions

Our study shows that reducing the procedure time can be the most effective factor an interventionist can modify to reduce thromboembolism after coil embolization of cerebral aneurysm. Additionally, our study shows that the longer procedure time may be affected by multiple procedural manipulations of each aneurysm and an inefficient periprocedural process of coil embolization. Our results suggest that clinical neurologists should refer patients with cerebral aneurysm to interventionists who have established an efficient procedural setting for coil embolization. Further study on this topic may lead to the development of a valuable factor of outcome for coil embolization.

## Ethics Statement

Because of study subject anonymity and minimal risk to the patients, collection of data without informed consent was approved by the local institutional review board of Chuncheon Sacred Heart Hospital before the study commenced.

## Author Contributions

S-HL: study design, clinical and image data acquisition, data interpretation, and primary writing; HC and J-HS: study design, clinical and image data acquisition, data interpretation, critical revision of the manuscript for important intellectual content, and supervision of the study; MJ, JK, YK, CK, JY, JJ, and YC: data acquisition and revision of manuscript.

### Conflict of interest statement

The authors declare that the research was conducted in the absence of any commercial or financial relationships that could be construed as a potential conflict of interest.
