# Supplementary material for: Impact of Reducing the Procedure Time on Thromboembolism After Coil Embolization of Cerebral Aneurysms
Source: Front Neurol. 2018 Dec 18;9:1125. doi: 10.3389/fneur.2018.01125 (PMC6305340; doi:10.3389/fneur.2018.01125)

Supplemental Table 1. Comparison of baseline characteristics between coiling-related and coiling-unrelated thromboembolism

|  | Thromboembolism  (-)  n=34 | Coiling-related thromboembolism  n=63 | Coiling-unrelated thromboembolism  n=83 | p-value |
| --- | --- | --- | --- | --- |
| **Patient factors** | | | | |
| Age (year), SD | 50.8±11. | 57.0±14. | 59.8±12.7 | 0.04^‡^ |
| Male sex, % | 210 (29.4) | 320 (31.7) | 37 (44.6) | 0.16^*^ |
| **Vascular risk factors** | | | | |
| History of using antiplatelet agents, % | 5 (14.7) | 9 (14.3) | 9 (10.8) | 0.52^*^ |
| Presence of hypertension, % | 7 (20.6) | 38 (60.8) | 31 (37.3) | 0.001^*^ |
| Presence of diabetes mellitus, % | 2 (5.9) | 7 (11.1) | 10 (12.0) | 0.61^*^ |
| Presence of coronary artery disease, % | 0 (0.0) | 5 (7.9) | 2 (2.4) | 0.10^†^ |
| Presence of hyperlipidemia, % | 2 (5.9) | 5 (7.9) | 5 (6.0) | 0.88^†^ |
| Current smoker, % | 10 (29.4) | 17 (27.0) | 24 (28.9) | 0.95^*^ |
| History of stroke, % | 12 (35.3) | 18 (28.6) | 21 (25.3) | 0.55^*^ |
| **Aneurysmal factors** | | | | |
| Size of the aneurysm, % |  |  |  | 0.98^*^ |
| ≤7 mm | 26 (76.5) | 49 (77.8) | 65 (78.3) |  |
| >7 mm | 8 (23.5) | 14 (22.2) | 18 (21.7) |  |
| Ruptured aneurysm, % | 22 (64.7) | 37 (58.7) | 50 (60.2) | 0.86^*^ |
| Aneurysm location, % |  |  |  | 0.13^*^ |
| MCA | 3 (8.8) | 10 (15.9) | 7 (8.4) |  |
| ACA | 15 (44.1) | 16 (25.4) | 41 (49.4) |  |
| ICA | 13 (38.2) | 30 (47.6) | 26 (31.3) |  |
| Posterior circulation artery | 3 (8.8) | 7 (11.1) | 9 (10.8) |  |
| Morphology of the aneurysm, % |  |  |  | 0.97^*^ |
| Fusiform | 8 (23.5) | 13 (20.6) | 18 (21.7) |  |
| Saccular | 26 (76.5) | 50 (79.4) | 65 (78.3) |  |
| **Procedure-related factors** | | | | |
| Simple coiling, % | 17 (50.0) | 31 (49.2) | 42 (50.6) | 0.99^*^ |
| Stent-assisted coiling, % | 13 (38.2) | 17 (27.0) | 29 (34.9) | 0.47^*^ |
| Use of multiple microcatheters, % | 4 (11.8) | 15 (23.8) | 12 (14.5) | 0.20^*^ |
| Number of coils inserted, n | 4.9±3.5 | 5.5±5.9 | 4.3±3.3 | 0.30^‡^ |
| Premedication with antiplatelet agents, % | 14 (41.2) | 28 (44.4) | 37 (44.6) | 0.96^*^ |
| Procedure time (min), SD | 92.7±37.2 | 122.3±62.4 | 109.0±62.2 | 0.06^*^ |
| **Laboratory results** | | | | |
| Hemoglobin level (mg/dl), SD | 13.4±1.6 | 17.6±22.8 | 13.7±1.7 | 0.18^‡^ |
| Platelet count (×1000/u), SD | 266.7±86.9 | 268.2±81.7 | 255.9±70.5 | 0.60^‡^ |
| Total cholesterol (mg/dl), SD | 171.7±30.5 | 181.5±47.2 | 179.2±36.6 | 0.50^‡^ |

SD, standard deviation; MCA, middle cerebral artery; ACA, anterior cerebral artery; ICA, internal carotid artery; BA, basilar artery; VA, vertebral artery

^*^ Calculated by the chi-square test

^†^ Calculated by Fisher’s exact test

^‡^ Calculated by analysis of variance

Supplemental Table 2. Multinomial regression analysis showing ORs of each procedure-related factor for the occurrence of coiling-related and coiling-unrelated thromboembolism (reference: DWI-negative group)

| **Coiling-related thromboembolism** | | | | | |
| --- | --- | --- | --- | --- | --- |
|  | Crude OR | 95% CI | Adjusted OR | 95% CI | p-value |
| Procedure time per 10-minute increase | 1.10 | 1.01-1.20 | 1.11 | 1.01-1.22 | 0.03 |
| Simple coiling | 0.97 | 0.42-2.23 | 1.07 | 0.45-2.58 | 0.88 |
| Stent-assisted coiling | 0.60 | 0.25-1.45 | 0.45 | 0.18-1.17 | 0.10 |
| Use of multiple microcatheters | 2.34 | 0.71-7.73 | 2.96 | 0.84-10.43 | 0.09 |
| Number of coils inserted | 1.03 | 0.94-1.12 | 0.997 | 0.90-1.10 | 0.96 |
| Premedication with antiplatelet agents | 1.14 | 0.49-2.66 | 0.90 | 0.37-2.21 | 0.82 |
| **Coiling-unrelated thromboembolism** | | | | | |
|  | Crude OR | 95% CI | Adjusted OR | 95% CI | p-value |
| Procedure time per 10-min increase | 1.06 | 0.98-1.16 | 1.08 | 0.99-1.18 | 0.10 |
| Simple coiling | 1.02 | 0.46-2.28 | 1.07 | 0.47-2.45 | 0.88 |
| Stent-assisted coiling | 0.87 | 0.38-1.98 | 0.76 | 0.32-1.80 | 0.53 |
| Use of multiple microcatheters | 1.27 | 0.38-4.25 | 1.45 | 0.41-5.14 | 0.56 |
| Number of coils inserted | 0.67 | 0.84-1.07 | 0.93 | 0.83-1.03 | 0.16 |
| Premedication with antiplatelet agents | 1.15 | 0.51-2.58 | 0.97 | 0.42-2.26 | 0.95 |

The multinomial regression analysis was adjusted for age, hypertension, aneurysmal size, and each procedure-related factor.

Supplemental figure 1. The DWI images of representative cases that had poor functional outcomes after coil embolization


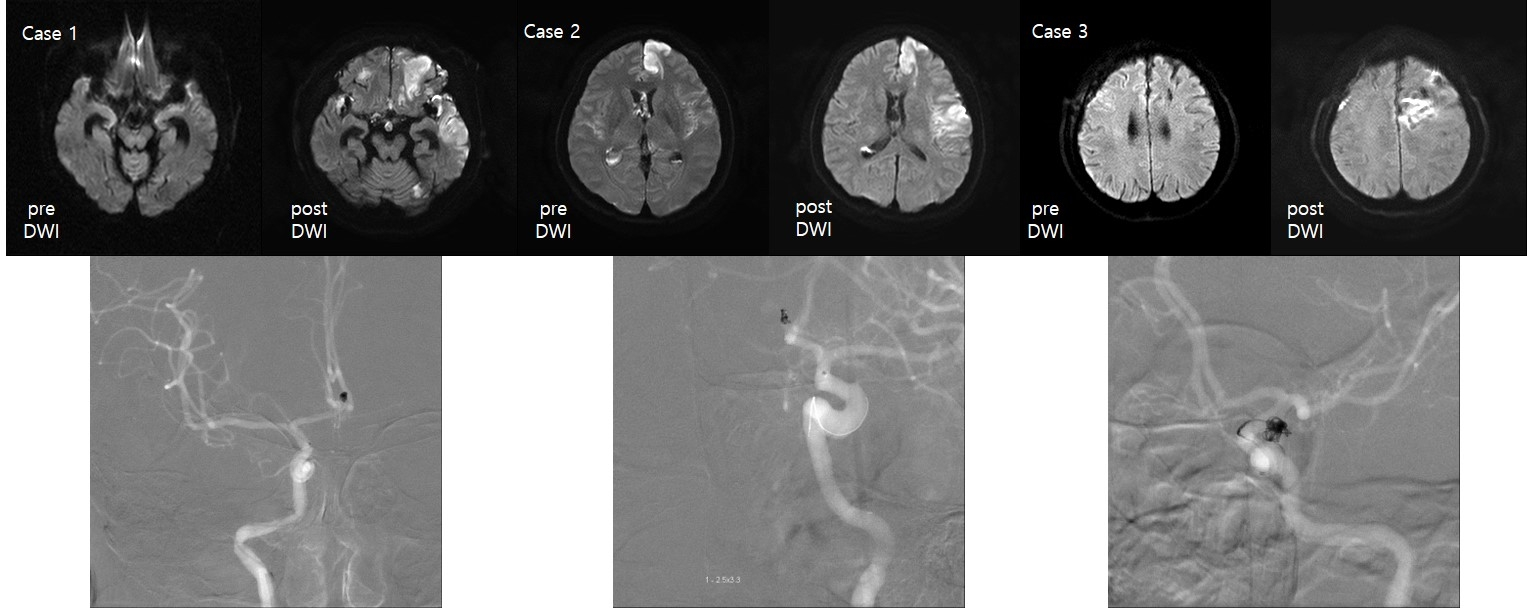

Supplement: Supplementary file 1 [file Data_Sheet_1.docx]
